# Health care transition from pediatric to adult care: an evidence-based guideline

**DOI:** 10.1007/s00431-022-04385-z

**Published:** 2022-01-27

**Authors:** Lars Pape, Gundula Ernst

**Affiliations:** 1 Society for Transition Medicine, Hannover, Germany; 2 Children’s Clinic II, Essen University Hospital, Univ. of Duisburg-Essen, Essen, Germany; 3 Research and Teaching Unit Medical Psychology, Hannover Medical School, Hannover, Germany

**Keywords:** Health care transition, Guideline, Evidence, Systematic review, Adolescents

## Abstract

For consistent quality of treatment of young people with chronic health care needs, a structured health care transition (HCT) from pediatric medicine to adult medical care is essential. Currently, most countries have no guidelines on HCT based on systematic evidence research. To close this gap, guidance to support HCT was developed in Germany. Twenty-two experts in the management of adolescents and young adults with different chronic conditions and three patient representatives were invited to take part in the guideline group. Based on a systematic literature search recommendations for HCT were drafted. Where evidence was lacking, recommendations were developed using collective expert consensus. The consensus process was independently moderated using a Delphi approach. The final draft was reviewed and endorsed by all major German medical societies. The clinical recommendations provide guidance for all chronic somatic diseases. After assessment of HCT readiness, an individualized plan for HCT should be drawn up. Key elements here are a responsible coordinator, age-adapted patient education, involvement of caregivers, web-based interventions, joint visits, and a structured summary for the receiving physician. The aim is the gradual transfer of responsibility for disease management to the young person themselves.

*   Conclusion*: As only a few randomized controlled studies on HCT are available, evidence-based statements are possible for some but not all areas of HCT. However, this guideline may help to develop globally accepted standards. These standards should be established and implemented. The aim should be a reimbursement by individual national health systems to allow appropriate support for young people.

**Table:** 

**What is Known:** *• Health care transition from pediatric to adult care is primarily organized in local settings, partly based on disease-specific guidelines.* *•There have been no national guidelines in Germany until now.*	
**What is New:** *• Here we present the general evidence-based guidelines of the German Association of Scientific Medical Societies for health care transition.* *• These might serve as a blueprint for further national or international health care transition guidelines.*

**Supplementary information:**

The online version contains supplementary material available at 10.1007/s00431-022-04385-z.

## Introduction

In Germany, 11.4% of girls and 16.0% of boys suffer from chronic diseases [1]. The health care transition (HCT), i.e., the preparation and follow-up of the transfer from pediatric to adult medicine, poses a challenge for these adolescents. Up to 40% of adolescent patients lose access to special care during the HCT from pediatric medicine to adult medical care [2]. There is a danger of undersupply of medical care and thus a risk to individual health from, for example, an increased rate of transplant losses and renewed dialysis in patients after kidney transplantation [3], reduced use of immunosuppressants in patients after liver transplantation [4], and a lack of specialized care in many young people with congenital heart defects [5], juvenile idiopathic arthritis [6], and diabetes [7], all of which lead to a significant impact on patient safety and health care costs [8].

In order to avoid such negative consequences for both the individual and for health-related expenditure, a structured and planned HCT process is necessary, as described in international recommendations on HCT [9, 10]. In addition to the medical aspects, this must also include psychosocial and professional features [11].

In the last few years, international consensus statements for a variety of diseases have been published [12–15], and both outcomes [16, 17] and models for research have been defined [18, 19]. There is currently a lack of standards for the HCT process and secure funding for it based on systematic evidence research [11, 20].

To close this gap, we developed an evidence-based guideline, which aims to create guidance for clinicians and other health professionals in their daily clinical practice when treating adolescents and young adults. In contrast to disease-specific consensus statements, it relates to all chronic somatic diseases.

## Methods

The guideline was developed in four stages according to the recommendations of the German Association of the Scientific Medical Societies (https://www.awmf.org/fileadmin/user_upload/Leitlinien/AWMF-Regelwerk/AWMF-Guidance_2013.pdf). This recommendation includes a complete risk bias assessment. Stage 1: Between 2018 and 2019 a systematic literature search was carried out by independent researchers using the PRISMA checklist to evaluate bias and the GRADE system to grade recommendations. The procedure and the results of the systematic review have already been published separately [21, 22]. The online supplements of these publications include tables describing the exact assessment of each study and possible bias, as well as a table explaining why bias has led to certain studies being excluded from the analysis. Overall, the 40 included studies assessed the outcomes of 3333 patients aged 12–28 years. Stage 2: Two coordinators from the Society for Transition Medicine (LP, GE) drafted a set of recommendations based on this systematic literature search. Stage 3: The evidence-based recommendations were presented as a word document to 20 additional experts in the management of adolescents and young adults with different chronic conditions and three members of self-help groups (Supplementary Table 1) and these recommendations were then discussed in an independently moderated workshop. The 19 experts have each been selected and named by the participating societies. Where evidence for important aspects of the HCT was lacking, the guideline group formulated additional recommendations based on collective expert opinion in the workshop. The guideline group was then invited to agree/disagree with the draft recommendations in an in-person discussion and by voting. Where there was disagreement, the coordinators reviewed the comments and made any necessary amendments. Stage 4: The revised guidelines were presented as a new word document and there were online re-votes for recommendations; this process continued until consensus was achieved on nearly all final recommendations using a Delphi approach. The grading of evidence, strength of recommendation, and consensus are outlined in Table 1.

**Table 1:**
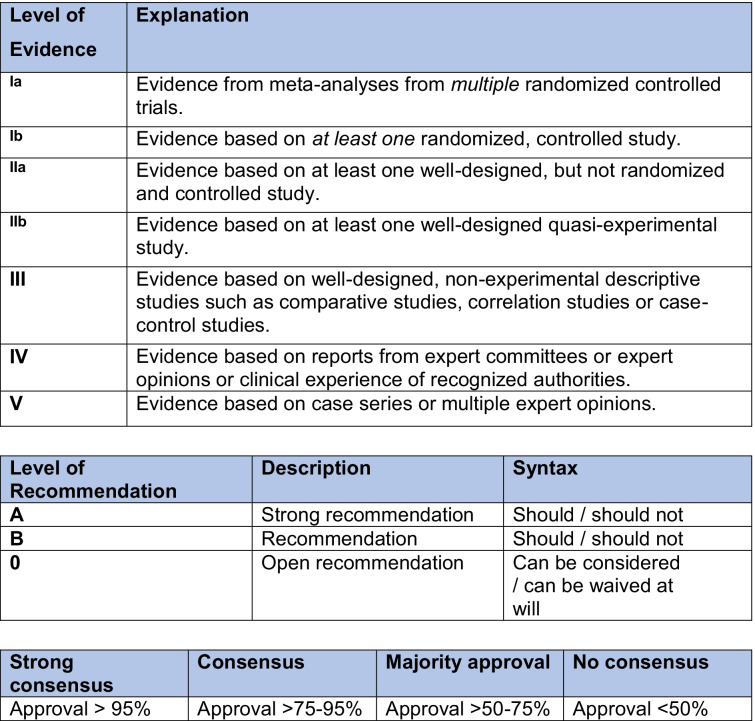
Grading of evidence, strength of recommendation, and consensus

The final draft was reviewed and endorsed by all major German medical societies and was accepted by the German Association of the Scientific Medical Societies after a review and a submission of a revised version that completely fulfilled the recommendations of the association on March 17, 2021.

## Results

### Recommendations for HCT with evidence

#### For the HCT process, an individualized plan should be created that defines and schedules the individual aspects of HCT (Level of Evidence II, Level of Recommendation B, strong consensus)

An HCT plan was tested as a central measure in two studies. A quasi-experimental study [23] found that young people with congenital heart defects had a faster and more reliable route to specialized adult care after using a planning tool than was the case before introducing this resource. In addition, they showed a more stable complaint score over the HCT period than the comparison group. Weitz et al. [24] found better kidney function and fewer rejection episodes after the introduction of a structured HCT plan that considered the individual situation of young people after kidney transplantation.

In the control group of the only randomized controlled trial (RCT) with a focus on the route to specialized care [25], no difference was found in young people with type 1 diabetes in the first year after transfer. In the second year, however, there was a significantly higher rate of follow-up appointments and specialized care.

#### The willingness and ability to make the HCT should be recorded in a detailed clinical discussion (Level of Evidence I, Level of Recommendation A, strong consensus)

An assessment of HCT readiness combined with individual training was tested in an RCT in adolescents with congenital heart defects [26, 27]. Compared to the control group receiving standard care, the intervention group showed significant improvements in disease knowledge, self-management, and HCT-specific skills after 6 months. Additionally, they made an appointment with an adult cardiologist more quickly. Other studies that recorded HCT readiness are not very meaningful due to their small sample size [26–28].

#### The time of transfer should take into account the characteristics of the disease and the patient and should not be rigidly linked to the 18th birthday milestone (Level of Evidence II, Level of Recommendation B, strong consensus)

A systematic review of reviews provides moderate evidence that transfer of young people in late adolescence or early adulthood can improve HCT outcomes and patient satisfaction [29]. But none of our included studies examined the flexibility of transfer time as a sole intervention. In most studies, the timing was linked to individual HCT planning [24, 30, 31]. In other studies, the time of transfer was determined by an HCT coordinator [32]. After such tailored treatment adolescents showed better kidney function and fewer rejections after kidney transplantation [24]. Cole and co-workers observed better drug adherence, greater use of the first appointment, fewer operations, and fewer hospital admissions in the first 2 years after transfer in young patients with inflammatory bowel disease [30]. In young people with type 1 diabetes the HbA1c value improved significantly [31]. Jensen and colleagues showed a higher rate of successful HCT in adolescents with juvenile idiopathic arthritis after complex care coordinated by a social worker [32].

#### The HCT process should include patient education for the patients and, if necessary, their caregivers on relevant aspects of the disease and transfer (Level of Evidence Ib, Level of Recommendation A, strong consensus)

Training measures as the main intervention for adolescents were examined in several studies. For example, adolescents with congenital heart defects were informed about their disease, potential risks, and HCT after an assessment of HCT-specific skills in a 1- or 2-h individual training course [26, 27]. Both RCTs showed positive effects on disease knowledge, self-management, and HCT-specific skills, as well as on the use of doctors’ appointments in the second study. For a day and a half youth-specific group education program, two prospective quasi-experimental studies also showed positive effects on patient activation, and HCT-specific knowledge and skills, but not on health-related quality of life [33–35].

#### The HCT process should have an interdisciplinary design (Level of Evidence II, Level of Recommendation B, strong consensus)

For young people with epilepsy, Geerlings et al. [36] set up an HCT consultation hour with a multidisciplinary team consisting of neurologists, neuropsychologists, social workers, and school/career counselors. After 1 year it was found that participation in the intervention was an important predictor of improvement in medical and academic outcomes. Yerushalmy-Feler and colleagues implemented a multidisciplinary HCT consultation hour for young people with inflammatory bowel disease, which offered adolescents the opportunity to speak with a pediatrician, an adult gastroenterologist, a nurse, and a psychologist. In the prospective pre-post comparison, a significant increase in self-efficacy could be demonstrated [37]. In more complex interventions, multidisciplinary teams were used to support the HCT process, for example, together with training courses or HCT coordinators [8, 30]. Consequently, the HCT team should consist of pediatric and adult-focused physicians, HCT coordinators, social workers, nurses, and psychologists.

#### A structured portable health summary on the previous course of the disease with medical and psychosocial content as well as treatment-relevant preliminary findings should be created for the patient and for further treatment (Level of Evidence II, Level of Recommendation B, strong consensus)

HCT summaries have been used as one aspect of very complex interventions [8], together with patient education programs [26] or an HCT coordinator [4]. Accordingly, the effect of a structured health summary or a patient passport is difficult to assess. In the study by Essaddam et al. [31] it was part of a joint HCT meeting for adolescents with type 1 diabetes. A patient card was filled out, which contained information on the patient’s history, medication, and complications, as well as providing a psychosocial background and professional perspective. In the prospective study, 75% of the participants showed metabolic improvements 1 year after the transfer.

#### A responsible person should accompany the young person during the transfer to adult-focused care (Level of Evidence II, Levels of Recommendation B, strong consensus)

The effect of a responsible contact person for the HCT was investigated in three controlled, but not randomized, studies. A social worker accompanied young people with juvenile ideopathic arthritis throughout the entire HCT period [32]. In the intervention group, the rate of adolescents successfully transferred to adult care was significantly higher than among adolescents who refused to participate in the program and who acted as a control group (42% vs. 23% with at least two doctor’s appointments in the follow-up period/15% vs. 58% without an appointment). In the only RCT in adolescents with diabetes, there were no demonstrable effects from telephone contact with a coordinator. Three short phone calls were made to the young people, who were asked about their well-being, any special events, and problems in the HCT process [38].

#### In order to improve adherence to treatment and appointments, low-threshold offers should be used through websites, apps, SMS, email, and/or telephone, if accessible (Level of Evidence II, Level of Recommendation B, strong consensus)

Three RCTs on the use of digital media to promote self-management in young patients [39–41] and one RCT on the use of telephone support were found [38].

Short telephone calls did not reveal any additional effects [38]. With the web-based schedules, the young people went through structured programs with weekly modules. All programs had additional personal support via telephone, SMS, or chat. The adolescents with hemophilia who were managed in this way had higher scores for disease-related knowledge, self-efficacy, and HCT readiness than control subjects [40]. Huang et al. [41] found a significant improvement in self-management and self-efficacy in adolescents with various chronic diseases.

#### In the case of younger adolescents, caregivers should be included in the HCT process. In the case of patients with cognitive impairments, the involvement of caregivers/permanent caregivers is mandatory (Level of Evidence III, Level of Recommendation A, strong consensus)

For ethical reasons, studies in adolescents that do not involve caregivers are not possible. In a longitudinal observational study, centers with different grades of parental involvement were compared with one another. One year after the transfer, it was found that the involvement of caregivers was strongly associated with the psychological well-being of the patient and with their satisfaction with the health services [42]. In the program by Menrath et al. [33] a youth-specific education program was expanded by the addition of a half-day training course for caregivers. Caregivers and adolescents were very satisfied with this course and the adolescents showed higher values for patient activation, HCT-specific knowledge, and competence after the workshop than controls. When the workshop was conducted without parental involvement, the value of HCT-specific competence rose less sharply [34].

#### The offer of a joint consultation or case conference, in which pediatricians and adult-focused physicians who provide further treatment are involved, can be considered (Level of Evidence III, Level of Recommendation 0, strong consensus)

Many studies test joint visits or case conferences mostly in interdisciplinary teams. These include intervention studies using a single-group design, sometimes with historical control groups for comparison [43–46]. In some cases, these are also retrospective comparisons of different groups [21, 36, 47]. Most of these studies found positive effects. Harden et al. [43] examined a small group of kidney-transplanted adolescents (*n* = 21) and observed fewer organ losses in the group that took part in the structured HCT program than in adolescents who were under care before the program was introduced (0 vs. 6). Levy-Shraga et al. [44] found significantly improved metabolic control in adolescents with diabetes and fewer diabetic ketoacidoses after the introduction of a joint clinic.

#### To support the HCT process, individual measures should not be used in isolation; instead, several of the elements described should be combined in a meaningful way (Level of Evidence II, Level of Recommendation B, strong consensus)

Almost all the studies were found to use several elements to support the HCT process, which can be explained by the interlinking of various interventions. Most studies that combined several HCT elements were able to show positive effects. In the few studies that only tested single elements [34, 38, 40, 47], this was only true in one case out of four.

### Recommendations for HCT based on expert consensus

#### Conversations about HCT should start early and in line with development of the adolescent (Level of Evidence IV, Level of Recommendation B, strong consensus)

From the beginning of adolescence, but no later than their 16th birthday, adolescents and, if necessary, their caregivers are advised on HCT-related topics in consultation hours and/or in separate education courses. An early start is necessary to pave the way for the transfer and to initiate any necessary measures.

#### In the HCT process, topics relevant to young people, such as sexuality, family planning, sleep–wake rhythms, consumption of alcohol, nicotine, and illegal substances and their interaction with the disease and its therapy, should be addressed by the treatment team (Level of Evidence IV; Level of Recommendation B, strong consensus)

The majority of adolescent patients have the same interests and needs as their healthy peers. However, they often find it difficult to reconcile their desire for a youthful lifestyle with the requirements of disease management. With a view to healthy psychosocial development, as well as enjoyment of a normal life, young people should be advised on how their needs can best be reconciled with their disease.

#### Screening for psychological stress and abnormalities should be part of the routine treatment for chronic illnesses (Level of Evidence IV, Level of Recommendation B, strong consensus)

In adolescence, young people become fully aware of the chronic nature and possible consequences of their disease. In addition, disease management and onerous disease-related restrictions can easily lead to frustration and self-doubt. The treatment team should sensitively explore insecurities about self-management, disturbed eating behavior, depression, and worries about the future, in addition to adherence problems and therapy fatigue.

#### Sufficient time should be planned for detailed HCT appointments within the pediatric service but also with the receiving doctor (Level of Evidence IV, Level of Recommendation B, strong consensus)

The counseling and training of the young patients and their caregivers requires a considerable amount of time within the care service, and it is therefore necessary to plan for longer appointments. It must be possible to account for this additional work appropriately.

#### The responsibility for disease management should gradually be transferred from caregivers to adolescents (Level of Evidence IV, Level of Recommendation B, strong consensus)

To avoid excessive demands and to slowly prepare both sides for their new roles, the responsibility for disease management should be gradually transferred from the caregivers to the adolescent. This process must be adapted to the developmental and cognitive capabilities of the adolescent and the complexity of the therapy.

#### Advice on professional and social issues related to the disease should be offered to young people (Level of Evidence IV, Level of Recommendation B, strong consensus)

Whether he or she is entitled to benefits under social law should be discussed with the young person, if appropriate. Current social law benefits must be checked and reapplied for. The patient should know contact points for further information.

#### Young people should be made aware of relevant self-help associations and patient organizations (Level of Evidence IV, Level of Recommendation B, strong consensus)

Self-help associations and patient organizations provide a variety of programs that strengthen HCT structures and processes. They advise both those affected and health care professionals, provide information specific to the disease, create platforms for the exchange of information, and offer individual support. In doing so, the personal competence of those affected is strengthened.

## Discussion

National German cross-disease recommendations for important HCT factors for which only partial evidence exists were established in this guideline with the broad participation and agreement of specialist societies. The recommendations seem consistent with published disease specific-recommendations [9, 10, 12–16, 20]. The novelty of our guideline is the cross-disease aspect, defining an HCT standard. Interestingly, there is a strong consensus for almost all recommendations despite often low levels of evidence. This underlines the fact that many strategies for HCT have been similarly established worldwide without complete evidence. In several areas where transition procedures have been established, it might even be contradictory to perform RCTs as it would be unethical to have a control group who would not receive an adequate HCT.

The recommendations will serve as a foundation for HCT in Germany for frequent as well as rare diseases. Obviously, disease-specific aspects will have to be added and there must be additional recommendations for young people with multiple disabilities, non-autonomous patients, and those with psychiatric diseases. The applicability will also be based primarily on the implementation of national HCT structures in Germany [11] and the reimbursement of the different elements of HCT. In addition, HCT should become a supplementary subject of education and board certification in pediatrics and adolescent health. To improve the quality of HCT in future, milestones for a stepwise implementation of the guidelines described here must be defined and controlled, and a clearly defined quality check for HCT will have to be implemented and linked to complete health-care reimbursement in this age group. HCT can be even further improved by providing an additional focus for the development of self-management skills, interventions to improve mental health, reduction of fear of the unknown related to the switch to adult care, and easier access to appropriately trained adult providers.

The recommendations of this guideline may serve as a blueprint for the national HCT guidelines of other countries. The recommendations given in this manuscript focus on Germany but most of them are broadly accepted worldwide. They can only be implemented if there is adequate funding to cover time, staff, and structures within the HCT services of national health systems. The implementation in other countries will depend on the structure of the individual national health care system and might be faster in those countries with a centralized system.

These guidelines have important limitations. Most of the actual recommendations for the HCT of young people are disease specific and not evidence based. Only a few high-quality studies, i.e., RCTs, are available on HCT [21, 22], so that evidence-based statements are possible for some, but not all, areas. Only three adult patient representatives and no adolescents/young adults themselves were involved in the Delphi consensus process. This could have strengthened the quality of the guidelines. The guidelines exclude mental health conditions, neurodevelopmental conditions, and psychiatric diseases as the corresponding German societies plan to develop their own guidelines on HCT. Due to the literature research process of the underlying review articles [21, 22] in this guideline, information on additional transition interventions, such as that from the literature on self-management, have not been considered here.

## Conclusion

This guideline, partly based on evidence, may help to develop globally accepted standards for HCT that should be established, implemented, and properly funded.

## Supplementary Information

Below is the link to the electronic supplementary material.

Supplementary file1 (DOCX 16 KB)

## Data Availability

All raw data is available from the authors.
