# Supplementary material for: Health care transition from pediatric to adult care: an evidence-based guideline
Source: Eur J Pediatr. 2022 Jan 27;181(5):1951–8. doi: 10.1007/s00431-022-04385-z (PMC9056438; doi:10.1007/s00431-022-04385-z)
Supplement: Supplementary file 1 — Supplementary file1 (DOCX 16 KB) [file 431_2022_4385_MOESM1_ESM.docx]

**Supplementary Table 1:** Members of the guideline group

| **Leading professional society** | |
| --- | --- |
| Gesellschaft für Transitionsmedizin (GfTM)  [Society of Transition Medicine] | Dr. Gundula Ernst, Hannover  Prof. Dr. Lars Pape, Essen |
| **Participating professional societies / associations / experts** | |
| Arbeitsgemeinschaft Pädiatrische Diabetologie  [Society of Pediatric Diabetology] | Dr. Thomas Kapellen, Bad Kösen |
| Deutsche Gesellschaft für Epileptologie (DGfE)  [German Society of Epileptology] | Dr. Frank Bösebeck, Rothenburg  Dr. Sarah von Spiczak, Kiel |
| Deutsche Gesellschaft für Innere Medizin (DGIM)  [German Society of Internal Medicine] | Prof. Dr. Britta Siegmund, Berlin |
| Deutsche Gesellschaft für Kinder- und Jugendmedizin (DGKJ)  [German Society of Pediatrics and Adolescent medicine] | Dr. Corinna Grasemann, Bochum  Prof. Dr. Hans Georg Koch, Braunschweig |
| Deutsche Gesellschaft für Kinderchirurgie  [German Society of Pediatric Surgery] | Prof. Dr. Jens Dingemann, Hannover |
| Deutsche Gesellschaft für Kinderendokrinologie und -diabetologie (DGKED)  [German Society of Pediatric Endocrinology] | Dr. Corinna Grasemann, Bochum |
| Deutsche Gesellschaft für Nephrologie  [German Society of Nephrology] | Prof. Dr. Lars Pape, Essen |
| Deutsche Gesellschaft für Palliativmedizin  [German Society of Palliative Care] | Christian Stellhorn, Wedemark |
| Deutsche Gesellschaft für Pneumologie und Beatmungsmedizin  [German Society of Pulmonology] | Dr. Christina Smaczny, Frankfurt |
| Deutsche Gesellschaft für **Psychosomatische** Medizin und Ärztliche Psychotherapie  [German Society of Psychosomatics and Psychotherapy] | Dr. Frank Vitinius, Köln |
| Deutsche Gesellschaft für Rheumatologie (DGRh)  [German Society of Rheumatology] | Dr. Susanne Schalm, München |
| Deutsche Gesellschaft für Sozialpädiatrie und Jugendmedizin (DGSPJ)  [Society of Social Pediatrics] | Dr. Helmut Peters, Mainz |
| Deutsches Kollegium für Psychosomatische Medizin (DKPM)  [German College of Psychosomatics] | Dr. Frank Vitinius, Köln |
| Gesellschaft für Kinder- und Jugendrheumatologie (DKJR)  [German Society of Pediatric Rheumatology] | Dr. Kirsten Minden, Berlin |
| Gesellschaft für Neuropädiatrie (GNP)  [German Society of Neuropaediatrics] | Dr. Ulrich Brandl, Jena  Dr. Regina Trollmann, Erlangen |
| Gesellschaft für Pädiatrische Nephrologie (GPN)  [German Society of Pediatric Nephrology] | Prof. Dr. Lars Pape, Essen |
| Gesellschaft für pädiatrische Onkologie und Hämatologie (GPOH)  [German Society of Pediatric Hematology and Oncology] | Prof. Dr. Holger Cario, Ulm  Dr. Gabriele Escherich, Hamburg |
| Berliner Transitionsprogramm (BTP)  [Berlin Transition Program] | Dr. Silvia Müther, Berlin |
| Kompetenznetz Patientenschulung im Kindes- und Jugendalter (KomPaS)  [Competence Net Patient Education] | Dr. Gundula Ernst, Hannover |
| Translate Namse  [National Association for Rare Diseases] | Dr. Corinna Grasemann, Bochum |
| TRANSNephro  [Transition in Nephrology] | Dr. Jenny Prüfe, Essen |
| Patientenvertreter - KEKS  [Patient focus group Esophageal Atresia] | Stephan Jechalke, Stuttgart |
| Patientenvertreter - Bundesvereinigung Jugendliche und Erwachsene mit angeborenen Herzfehlern (JEMAH)  [Patient focus group inborn heart diseases] | Roland Keuchen, Berlin |
| Patientenvertreter – Deutsche Rheuma-Liga Bundesverband  [Patient focus group Rheumatology] | Monika Mayer, Bonn |
